# circ_ZFR Is Linked to Paclitaxel Resistance in Cervical Cancer via miR-944 Sponging and IL-10 Upregulation

**DOI:** 10.1155/2022/4807287

**Published:** 2022-01-27

**Authors:** Xiaoqian Long, Meiyun Zheng, Youlin Yang, Yi Chen, Xiahui Zhang, Haiyan Zhang

**Affiliations:** Department of Obstetrics and Gynecology, The First People's Hospital of Wenling, Wenling, 317599 Zhejiang, China

## Abstract

**Objective:**

Cervical cancer (CC) has an elevated rate of invasion and death despite surgical treatment, radiotherapy, and chemotherapy. Several studies revealed that circRNAs have a key contribution to the resistance of drugs against different types of carcinomas. The goal of the existing study was to figure out what role circ_ZFR plays in paclitaxel (PTX) resistance in cervical cancer (CC) patients.

**Materials and Methods:**

Herein, two types of CC cells (SiHa/PTX and Hela/PTX) were utilized. The levels of IL-10 mRNA, miR-944, and circ_ZFR were measured using qRT-PCR analyses. The CCK-8 assay was used to determine PTX resistance. The IL-10 expression was measured via the ELISA technique. The combination of miR-944 and circ_ZFR or IL-10 was validated using a dual-luciferase reporter (DLR) assay.

**Results:**

The amount of circ_ZFR was increased in PTX-resistant CC cells and tissues. In PTX-resistant CC cells, knocking down circ_ZFR expression decreased PTX resistance. circ_ZFR knockdown significantly reduced IL-10 expression via sponging miR-944, increasing PTX sensitivity in PTX-resistant CC cells.

**Conclusion:**

circ_ZFR knockdown has a considerable role in overwhelming CC-associated PTX resistance by modifying the axis of miR-944/IL-10 axis, suggesting that developing a circRNA target-based treatment could be considered prevent CC progression.

## 1. Introduction

According to the global survey of cancer statistics, cervical cancer (CC) is considered to be the fourth most prevalent malignancy in women despite the HPV (human papillomavirus) vaccination and testing [1, 2]. CC has an elevated rate of invasion and death despite surgical treatment, radiotherapy, and chemotherapy. For resistant or recurring CC, the antitumor drug paclitaxel (PTX) is now commonly utilized, but PTX resistance has a significant impact on its therapeutic benefit [3]. As a result, enhancing clinical treatment methods requires extensive knowledge of the molecular mechanisms underlying PTX resistance in CC.

circRNAs are a type of unique noncoding RNA [4, 5] without 3′ and 5′ ends. Both ends are covalently connected which provides a circular shape to the RNA molecule [6, 7]. circRNAs were originally considered as splicing defects [8]; however, numerous studies have shown that these RNAs with abnormal expression have a key role in the advancement of various malignancies [9–12], including CC [13, 14]. circRNAs have been shown to influence chemoresistance [15–17]. circ_ZFR (also known as hsa-circ 0072088) promotes CC progression by Rb phosphorylation and through regulating the SSBP1/CDK2/cyclin E1 complex [18]. However, the implications of circ_ZFR for the CC's PTX resistance are remaining elusive.

The critical role of the circRNA-miRNA-mRNA regulation network in chemoresistance has been gradually explored [19–21]. According to our obtained data, miR-944 has the same binding sequences as circ_ZFR and IL-10 in the existing study, suggesting that circ_ZFR could act as a sponge for miR-944 to boost IL-10 expression. As a result, we decided to evaluate the implications of the circ_ZFR/miR-944/IL-10 axis for CC PTX resistance.

## 2. Materials and Methods

### 2.1. Tissue Acquisition

At Wenling First People's Hospital, 96 CC tissue samples (62 cervical squamous cell carcinoma and 34 cervical adenocarcinoma) and surrounding healthy tissue specimens were taken from patients associated with CC, followed by classification into two groups, i.e., the treatment-resistant group (*n* = 50) and the treatment-sensitive (*n* = 46) group. The existing study was authorized by the Wenling First People's Hospital Ethics Committee. All of the patients who took part in this study signed a written informed consent form.

### 2.2. Cell Proliferation and Cell Transfection

Human CC cell lines (SiHa and HeLa) were provided by the Shanghai Institutes for Biological Sciences (SIBS), China. The underlined cell was grown in liquid HyClone DMEM (Logan, UT, USA) along with 10% FBS. The culture mixtures were incubated (at 37°C) in the presence of air enriched with 5% CO_2_. HeLa and SiHa cell lines were cultured with the concentration of PTX increased from 10 to 20 *μ*g/L and 40 *μ*g/L. After 10 months of HeLa and SiHa cell lines' continuous culturing, PTX-resistant Hela/PTX and SiHa/PTX cell lines could be collected which maintained a good growth state in 40 *μ*g/L PTX. The SiHa/PTX and Hela/PTX cells were seeded in 6-well plates and incubated for 24 hrs before being treated with 50 nM of appropriate synthetic oligonucleotides or victor transfection (2 *μ*g) using Lipofectamine 2000 (11668500; Invitrogen), as the manufacturer recommended.

### 2.3. qRT-PCR Analyses

Following the manufacturer's recommendation, TRIzol (Invitrogen) was utilized to obtain total-RNA from CC cells or tissues. The TaqMan miRNA experiment was carried out to assess the amount of miR-944 (Thermo Fisher Sci, Inc., Waltham, MA, USA). The cDNA was extracted from 1 *μ*g of obtained RNA to evaluate the expression level of IL-10 and circ_ZFR by employing the Prime-Script-RT reagent Kit with gDNA remover (Takara, Dalian, China). 10 *μ*L of Go-Taq-qPCR Master Mix (Promega, Madison, WI, USA) was used to perform qRT-PCR analyses. In the existing study, we used U6 and GAPDH as reference genes. The 2^−*ΔΔ*CT^ approach was followed to analyze the obtained data. To assess the miR-944 expression, reverse transcription was carried out via a particular stem-loop RT-primer: 5′-CTCAACTGGTGTCGTGGAGTCGGCAATTCAGTTGAGCTCATCCG-3′.

The nucleotide sequences of the primer against circ_ZFR, IL-10, U6, and GAPDH have been shown as follows: circ_ZFR forward (F), 5′-AACCACCACAGATTCACTAT-3′ and reverse (R), 5′-AACCACCACAGATTCACTAT-3′; IL-10 F, 5′-GCCAAGCCTTGTCTGAGATGATCC-3′ and R, 5′-TTCACATGCGCCTTGATGTCTGG-3′; U6 F, 5′-TGCGGGTGCTCGCTTCGGCAGC-3′ and R, 5′-CCAGTGCAGGGTCCGAGGT-3′; and GAPDH F, 5′-CATGAGAAGTATGACAACAGCCT-3′ and R, 5′-AGTCCTTCCACGATACCAAAGT-3′.

### 2.4. PTX IC_50_ Assay

Using 96-well plates, 2 × 10^3^ transfected Hela/PTX and SiHa/PTX cells were grown and were then treated with different PTX concentrations (0.1, 1, 10, 100, and 1000 *μ*g/L). After 48 hrs, the seeded cells were treated with the CCK-8 solution (10 *μ*L), followed by thoroughly mixing them for 2 hrs. After the treatment of the CCK-8 solution, the O.D. (at 450 nm) of each well was recorded via an automated Multiskan-mk3 microplate reader (Thermo Fisher, USA).

### 2.5. Dual-Luciferase Reporter (DLR) Assay

The complete or mutant segments of the circ_ZFR or IL-10 3′UTR comprising the expected miR-944 interacting sites were cloned downstream of the luciferase gene in the pGL3 vector (Promega, Fitchburg, WI, USA). The cotransfection of 293 T cells was carried out with miR-NC or miR-944 in combination with IL-10 3′UTR-MUT or IL-10 3′UTR-WT or circ_ZFR-MUT or circ_ZFR-WT. After 24 hrs of the incubation period, luciferase activity was assessed using the DLR assay.

### 2.6. RNA Immunoprecipitation (RIP) Assay

The RIP assay was carried out with the help of the Magna RIP kit (Millipore). On the basis provided instructions (user's manual), Hela/PTX and SiHa/PTX cell transfection was carried out with miR-944 or miR-NC and cultured for 48 hrs before being lysed with RIP buffer having IgG-antibody or Ago2-labeled magnetic beads. Following that, TRIzol reagent was utilized to obtain immunoprecipitated RNAs. Furthermore, the circ_ZFR enrichment was determined via qRT-PCR evaluations.

### 2.7. RNA Pull-Down Assay

SiHa/PTX and Hela/PTX cell transfection was carried out via biotinylated circ_ZFR-MUT or ZFR-WT or NC, followed by 48 hrs of incubation. The cells were collected, lysed with RIP buffer, and then treated with streptavidin magnetic beads (M-280; Invitrogen) according to the manufacturer's instructions. The beads were thricely rinsed via a cold buffer after being incubated in a cold chamber (4°C) for two hours. Eventually, the RNA elution was carried out so that it could be used in other experimental evaluations.

### 2.8. ELISA

Following the instructions of the provider, an ELISA kit (R & D Systems) was employed for the measurement of IL-10 expression in various culture media. The average value was calculated from three experiments evaluated separately.

### 2.9. Statistics Analysis

The obtained data was compiled using SD from triplicated expressions (individually performed). GraphPad Ver 7.04 (La Jolla, CA, USA) was employed for the statistical evaluations on the data obtained. The means of continuous outcome variables were examined via ANOVA. Kaplan–Meier plots and log-rank test were conducted for the analysis of survival rates and to investigate variations among subgroups of patients. Variations were regarded as statistically considerable when the *p* values were less than 0.05.

## 3. Results

### 3.1. An Elevated Expression of circ_ZFR in CC Tissues and Cell Lines (Resistant to PTX)

**Figure 1:**
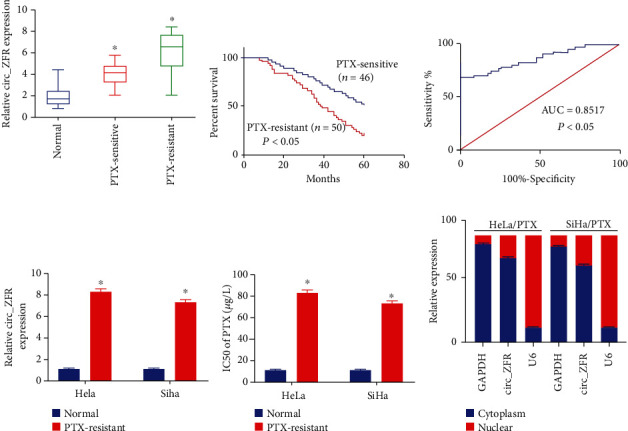
The overexpression of circ_ZFR in CC cells and tissues (PTX-resistant). (a) The qRT-PCR evaluations of circ_ZFR expression in CC tissues. (b) The survival rate in CC patients. (c) The ROC curve analysis of circ_ZFR for CC resistance. (d) The data of circ_ZFR expression obtained from the qRT-PCR assay. (e) The PTX IC_50_ values. (f) circ_ZFR expression in the nuclear and cytoplasm with ^∗^*p* < 0.05.

The circ**_**ZFR expression was evaluated in PTX-sensitive and PTX-resistant CC tissues *via* qRT-PCR analysis. The obtained data suggested that circ_ZFR expression was found to be elevated in CC tissues (resistant to PTX) in comparison with the healthy tissues and CC tissues (sensitive to PTX), as represented in Figure 1(a). Patients who were PTX-sensitive had a significant survival rate when treated with PTX, as indicated in Figure 1(b). The results of ROC curves showed that circ_ZFR may have diagnostic significance for PTX-resistant CC patients (Figure 1(c)). Furthermore, in HeLa and SiHa cells, the level of circ_ZFR was decreased relative to HeLa/PTX and SiHa/PTX cells, as depicted in Figure 1(d). The results obtained from the CCK-8 assay showed PTX resistance in HeLa/PTX and SiHa/PTX cells, as revealed by the higher IC_50_ concentration of PTX in HeLa/PTX and SiHa/PTX cells, as depicted in Figure 1(e). circ_ZFR was mostly found in the cytoplasmic part of the underlined cells, as indicated in Figure 1(f). The underlined data revealed that dysregulated circ_ZFR may have a role in the chemoresistance of CC.

### 3.2. Suppression of PTX Resistance in CC Cells (Resistant to PTX) by circ_ZFR Knockdown

**Figure 2:**
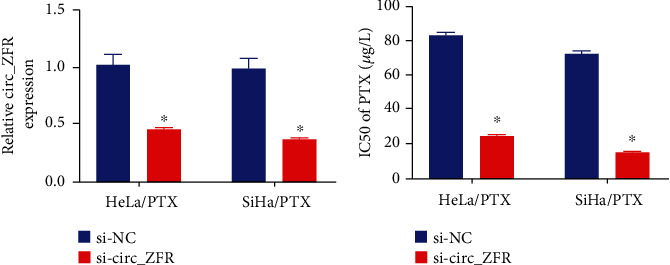
The suppression level of PTX resistance in circ_ZFR knockdown PTX-resistant CC cells. (a) The level of circ_ZFR expression in SiHa/PTX and HeLa/PTX cells. (b) The IC_50_ values of PTX for SiHa/PTX and HeLa/PTX cells with ^∗^*p* < 0.05.

To determine the role of circ_ZFR in PTX-resistant CC cells, si-circ_ZFR was transfected into HeLa/PTX and SiHa/PTX cells to knock down the circ_ZFR expression, and relative to si-NC groups, a considerable decrease was observed in the expression of circ_ZFR in HeLa/PTX and SiHa/PTX cells, as suggested by the qRT-PCR (Figure 2(a)). PTX IC_50_ value was lowered in the underlined cells (transfected with si-circ_ZFR), as indicated by CCK-8 assay which suggested that circ_ZFR knockdown overcomes PTX resistance in the underlined cells, as depicted in Figure 2(b). In PTX-resistant CC cells, knocking down circ_ZFR boosted the sensitivity of PTX.

### 3.3. Attenuation of miR-944 Enhanced the Impact of circ_ZFR Knockdown on PTX Sensitivity in CC Cells (Resistant to PTX)

**Figure 3:**
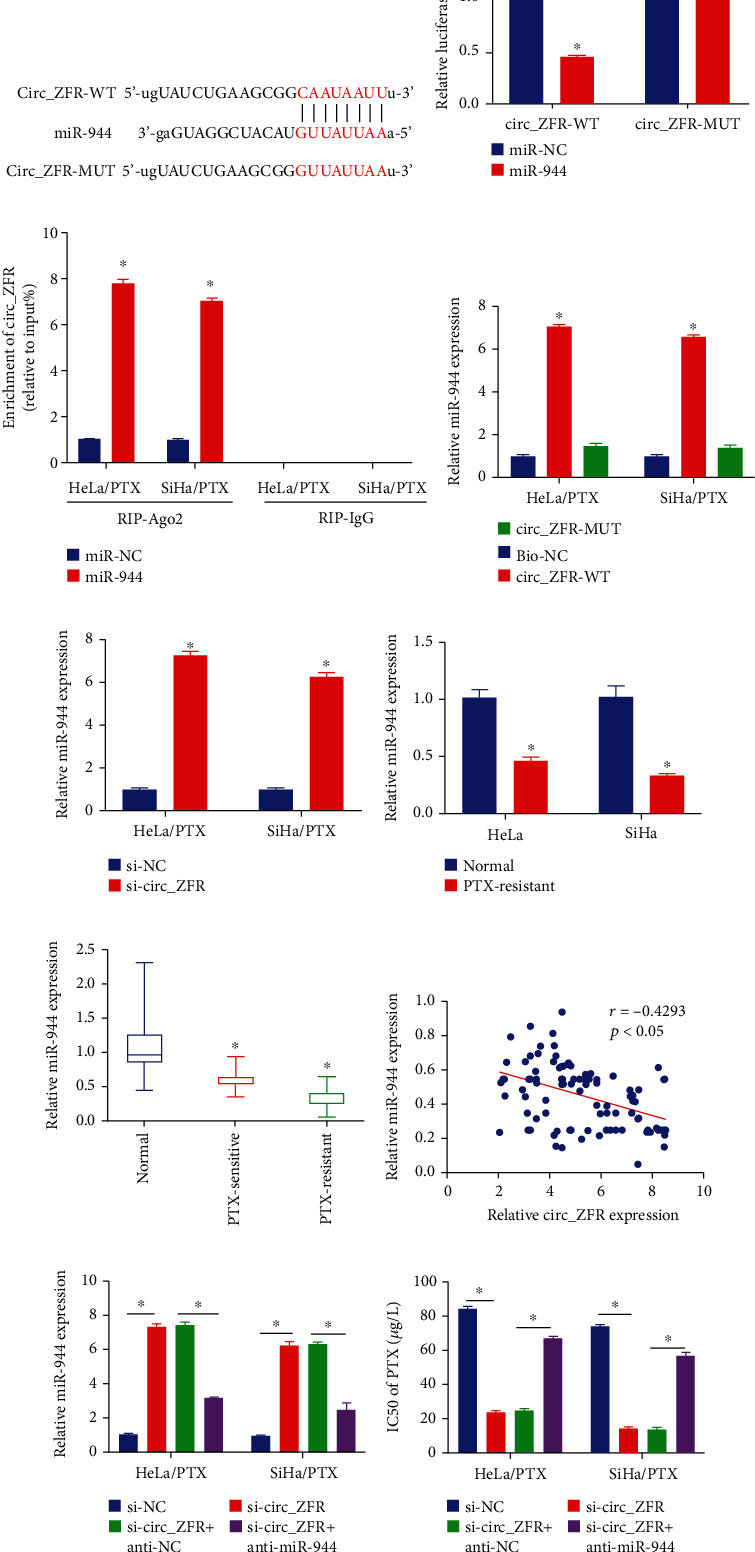
The miR-944 inhibition reduced the impact of circ_ZFR knockdown in PTX-resistant CC cells. (a) The possible interacting domains for miR-944 and circ_ZFR. (b) The interaction between miR-944 and circ_ZFR evaluated by DLR assay. (c, d) The data obtained from RIP and RNA pull-down assays accordingly. (e, f) The qRT-PCR data to evaluate the expression of miR-944. (g) The miR-944 expression in CC tissues. (h) The linkage between miR-944 and circ_ZFR. (i) The miR-944 expression level in SiHa/PTX and HeLa/PTX cell lines. (j) The PTX IC_50_ values in SiHa/PTX and HeLa/PTX cells. ^∗^*p* < 0.05.

Online tool starbase 3.0 was employed to examine the possible target of circ_ZFR to explore the pathway associated with circ_ZFR to regulate PTX resistance and cellular progression in CC cells (PTX resistant). miR-195-5p could be a circ_ZFR target, as it had the binding sites of circ_ZFR (Figure 3(a)). The DLR assay revealed that miR-994 transfection significantly reduced circ_ZFR-WT luciferase activity while not affecting circ_ZFR-MUT luciferase activity in 293 T cells, validating the link between circ_ZFR and miR-944, as depicted in Figure 3(b). Anti-AGO2 antibody was used to enrich endogenous circ_ZFR (Figure 3(c)). In HeLa/PTX and SiHa/PTX cells, the wild-type circ_ZFR collected more miR-944 than the mutant revealed by RNA pull-down assay (Figure 3(d)), showing that circ_ZFR directly targets miR-944. Furthermore, we discovered that silencing circ_ZFR increased the expression of miR-944 in the underlined cells, as depicted in Figure 3(e). The expression of miR-944 was elevated in HeLa and SiHa cells relative to HeLa/PTX and SiHa/PTX cells, as depicted in Figure 3(f). Furthermore, the miR-944 level decreased from normal tissues to CC tissues (PTX-sensitive) and then to CC tissues (PTX-resistant), as indicated in Figure 3(g). It has also been depicted that there was a negative association, existed between miR-944 and the circ_ZFR expression in tumor tissues (Figure 3(h)). miR-944 was negatively regulated by circ_ZFR through directly targeting. Based on the above findings, rescue experiments were conducted to evaluate whether PTX resistance has been regulated by circ_ZFR through miR-944 targeting. An elevated expression of miR-944 (inducted by si-circ_ZFR) was found to be reversed via anti-miR-944 transfection in HeLa/PTX and SiHa/PTX cells, as indicated in Figure 3(i). By lowering miR-944, the inhibitory effects of circ_ZFR knockdown on PTX resistance were all reversed in HeLa/PTX and SiHa/PTX cells (Figure 3(j)). The underlined results indicated that through sponging miR-944, circ_ZFR knockdown suppressed PTX-resistant CC cells.

### 3.4. An Elevated Expression of miR-944 Improved PTX Sensitivity in CC Cells (PTX-Resistant) by IL-10 Targeting

**Figure 4:**
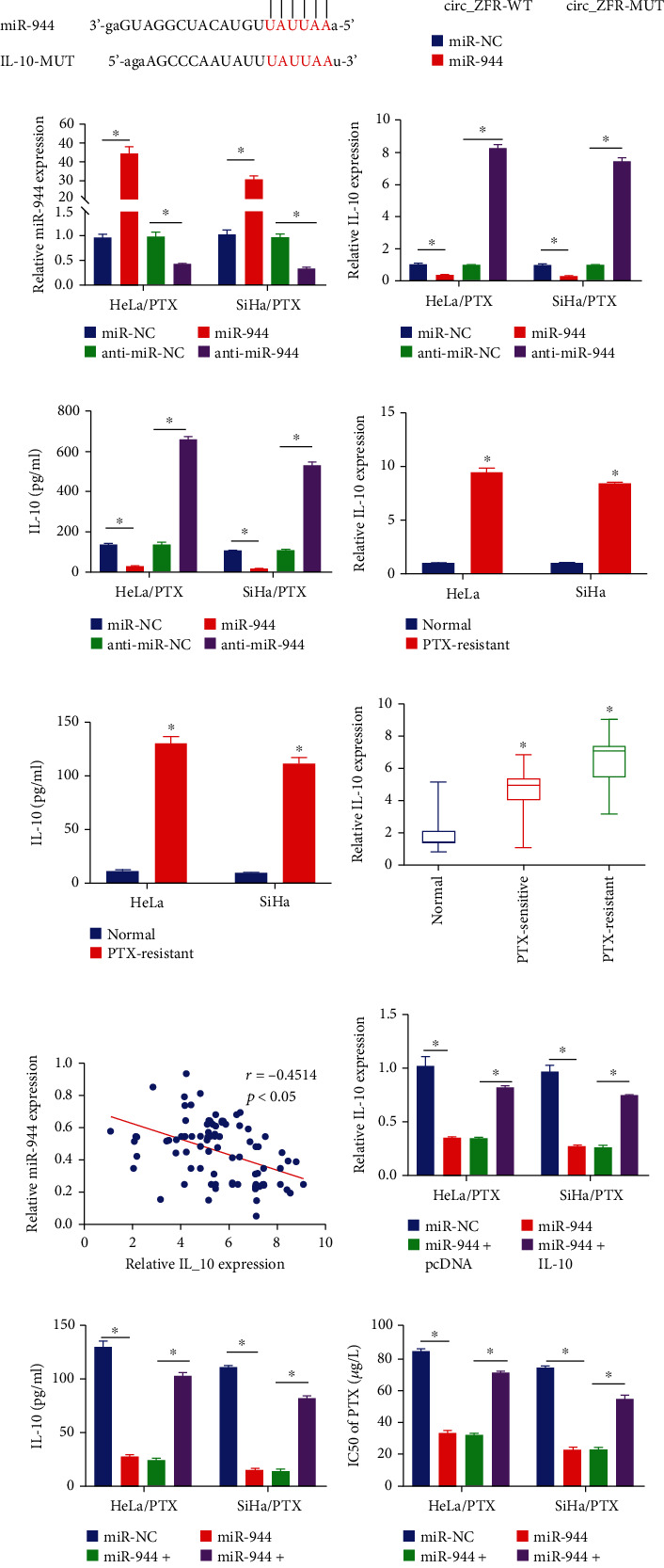
The targeting of IL-10 in PTX-resistant CC cells results in the overexpression of miR-944 and elevates PTX sensitivity. (a, b) The significant interaction sites between miR-944 and IL-10 determined by the DLR assay. (c) miR-944 expression was assessed via qRT-PCR. (d, f, j) The data obtained from qRT-PCR analysis to examine the expression level of IL-10. (e, g, k) The ELISA assay data to detect the expression level of IL-10. (h) The qRT-PCR evaluations for IL-10 level in CC tissues. (i) The correlation between IL-10 and miR-944 was estimated by Spearman's correlation coefficient analysis. (l) The IC_50_ values of PTX in HeLa/PTX and SiHa/PTX cells. ^∗^*p* < 0.05.

IL-10 was identified to be a target gene of miR-944 using starbase 3.0, and their possible interacting sites are shown in Figure 4(a). Then, using a DLR assay, it was discovered that in miR-944 and IL-10-WT cotransfected 293 T cells; luciferase activity was decreased in comparison to miR-NC and IL-10-WT cotransfected groups, but variations were not observed in IL-10-MUT groups (Figure 4(b)). To determine the impact of miR-944 on the expression of IL-10, miR-944, and anti-miR-944, their controls were transfected. HeLa/PTX and SiHa/PTX cells were successfully transfected with miR-944 and anti-miR-944, as shown in Figure 4(c). Furthermore, we evaluated that elevated expression of miR-944 considerably lowered the expression of IL-10 in HeLa/PTX and SiHa/PTX cells but attenuation of miR-944 showed apposing results (Figures 4(d) and 4(e)). Furthermore, our findings revealed that IL-10 expression was decreased in HeLa and SiHa cells relative to HeLa/PTX and SiHa/PTX cells, as depicted in Figures 4(f) and 4(g). From healthy tissues to tumor tissues (PTX-sensitive) and subsequently to tumor tissues (PTX-resistant), the expression of IL-10 was elevated (Figure 4(h)). The level of IL-10 mRNA in CC tissues was inversely linked with miR-944 expression using Spearman's correlation coefficient analysis, as revealed in Figure 4(i). miR-195-5p transfection significantly reduced IL-10 expression in HeLa/PTX and SiHa/PTX cells, as depicted in Figures 4(j) and 4(k), whereas the effects were restored by IL-10 transfection. miR-944 elevated expression successfully decreased PTX resistance in HeLa/PTX and SiHa/PTX cells using the CCK-8 assay, while elevated IL-10 successfully reversed the results (Figure 4(l)). In other words, elevated expression of miR-944, which targets IL-10, plays a considerable role in PTX sensitivity in CC cells (PTX-resistant).

### 3.5. circ_ZFR Knockdown Lowered the IL-10 Expression through Sponging miR-944

**Figure 5:**
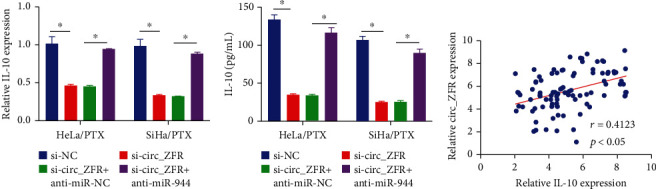
The circ_ZFR knockdown reduced the expression level of IL-10 via miR-944 sponging. (a, b) The data obtained from qRT-PCR and ELISA assay to detect the IL-10 expression. (c) The association between IL-10 and circ_ZFR. ^∗^*p* < 0.05.

For further investigation of the relationships between circ_ZFR, miR-944, and IL-10, the transfection of HeLa/PTX and SiHa/PTX cells was carried out with si-NC, si-circ_ZFR, si-circ_ZFR+anti-miR-NC, or si-circ_ZFR+anti-miR-944. In HeLa/PTX and SiHa/PTX cells, silencing of circ_ZFR significantly reduced IL-10 expression, as demonstrated in Figures 5(a) and 5(b), while miR-944 attenuation completely reversed the effects. The IL-10 mRNA level in CC tissue was revealed to be positively linked with the expression of circ_ZFR using Spearman's correlation coefficient analysis, as depicted in Figure 5(c). As a result, we concluded that circ_ZFR favorably controlled IL-10 expression in CC cells (PTX-resistant) via sponging miR-944.

## 4. Discussion

Chemoresistance is a major obstacle in the treatment of human malignancies, including CC. Various circRNAs have been confirmed to have a role in regulating the development of chemoresistance [22, 23]. The existing study is aimed at seeing what role circ_ZFR plays in modulating CC's PTX sensitivity. As a result of the modulation of the miR-944/IL-10 axis by circ_ZFR, PTX resistance in CC cells (PTX-resistant) was facilitated.

The importance of circRNAs in tumor growth and treatment resistance has steadily drawn researchers' attention in CC. circMTO1, for example, promotes cervical cancer growth and chemoresistance by miR-6893 regulation [21]. hsa-circ 0023404 sponging miR-5047 elevated progression and chemoresistance in cervical cancer via VEGFA and autophagy signaling [24]. The underlined findings revealed that circRNAs performed a variety of roles in CC drug resistance. circ_ZFR has a role in the paclitaxel resistance and development of NSCLC by elevated expression of KPNA4 through sponging miR-195-5p [25]. By modulating the miR-545-3p/CBLL1 axis, circ 0072083 interference suppressed DDP resistance, formation of cell colony, cell cycle, and metastasis and accelerated the apoptotic process in NSCLC cells [26]. In this study, a high level of circ_ZFR was found in chemoresistant CC tissues and cells. In vitro, circ_ZFR deficiency increased PTX sensitivity in CC cells (resistant to PTX). circ_ZFR has a significant contribution to PTX resistance in CC as a whole.

circ_ZFR was evaluated as the sponge for miR-944, which then boosted IL-6 expression, according to the mechanistic analysis. The miR-944/FZD7 axis attenuated doxorubicin resistance and reduced tumor growth in colorectal cancer when the circular RNA circCSPP1 was knocked down [27]. The obtained results revealed that miR-944 appeared to have a considerable contribution to the development and chemoresistance of CC. Our findings suggested that miR-944 levels were lowered in CC tissues and cells (resistant to PTX). The impact of circ_ZFR knockdown on PTX sensitivity may be effectively restored by suppressing miR-944 in CC cells (PTX-resistant). Furthermore, elevated expression of miR-944 boosted the sensitivity of PTX in cells (PTX-resistant); however, these effects might be reversed by an elevated expression of IL-10. Based on the obtained results, miR-944 decreased PTX resistance in CC cells (PTX-resistant) by targeting IL-10. In support of our results, AS101 sensitizes tumors to chemotherapy by blocking the tumor IL-10 autocrine loop which supports our findings [28]. Although IL-10 is a target of a variety of miRNAs, including miR-374b-5p [29] and miR-98 [30], we are the first to show an interaction between miR-944 and IL-10 in CC chemoresistance.

In conclusion, the obtained data from the existing study revealed that circ_ZFR enhanced PTX resistance in CC by modulating the miR-944/IL-10 axis, which may have a candidate therapeutic strategy to decrease the resistance of CC to PTX.

## Data Availability

Due to the nature of this research, participants of this study did not agree for their data to be shared publicly, so supporting data is not available.
